# Root Exposure to 5-Aminolevulinic Acid (ALA) Affects Leaf Element Accumulation, Isoprene Emission, Phytohormonal Balance, and Photosynthesis of Salt-Stressed *Arundo donax*

**DOI:** 10.3390/ijms23084311

**Published:** 2022-04-13

**Authors:** Federico Brilli, Sara Pignattelli, Rita Baraldi, Luisa Neri, Susanna Pollastri, Cristina Gonnelli, Alessio Giovannelli, Francesco Loreto, Claudia Cocozza

**Affiliations:** 1 Institute for Sustainable Plant Protectio, National Research Council of Italy (IPSP-CNR), Via Madonna del Piano 10, 50019 Sesto Fiorentino, Italy; sara.pignattelli@ibbr.cnr.it (S.P.); susanna.pollastri@ipsp.cnr.it (S.P.); francesco.loreto@unina.it (F.L.); claudia.cocozza@unifi.it (C.C.); 2 Institute of Biosciences and BioResources, National Research Council of Italy (IBBR-CNR), Via Madonna del Piano 10, 50019 Sesto Fiorentino, Italy; 3 Institute for BioEconomy, National Research Council of Italy (IBE-CNR), Via Gobetti 101, 40129 Bologna, Italy; rita.baraldi@ibe.cnr.it (R.B.); luisa.neri@ibe.cnr.it (L.N.); 4 Department of Biology, University of Florence, Via Micheli 1, 50121 Firenze, Italy; cristina.gonnelli@unifi.it; 5 Research Institute on Terrestrial Ecosystems, National Research Council of Italy (IRET-CNR), Via Madonna del Piano 10, 5001 Sesto Fiorentino, Italy; alessio.giovannelli@cnr.it; 6 Department of Biology, University of Naples “Federico II”, Via Cinthia 7, 80126 Napoli, Italy; 7 Department of Agriculture Food Environment and Forestry, University of Florence, Via San Bon-Aventura 13, 50145 Firenze, Italy

**Keywords:** volatile organic compounds (VOCs), indol-3-acetic acid (IAA), abscisic acid (ABA), growth, sodium (Na^+^), stress tolerance

## Abstract

*Arundo donax* has been recognized as a promising crop for biomass production on marginal lands due to its superior productivity and stress tolerance. However, salt stress negatively impacts *A. donax* growth and photosynthesis. In this study, we tested whether the tolerance of *A. donax* to salinity stress can be enhanced by the addition of 5-aminolevulinic acid (ALA), a known promoter of plant growth and abiotic stress tolerance. Our results indicated that root exposure to ALA increased the ALA levels in leaves along the *A. donax* plant profile. ALA enhanced Na^+^ accumulation in the roots of salt-stressed plants and, at the same time, lowered Na^+^ concentration in leaves, while a reduced callose amount was found in the root tissue. ALA also improved the photosynthetic performance of salt-stressed apical leaves by stimulating stomatal opening and preventing an increase in the ratio between abscisic acid (ABA) and indol-3-acetic acid (IAA), without affecting leaf methanol emission and plant growth. Supply of ALA to the roots reduced isoprene fluxes from leaves of non-stressed plants, while it sustained isoprene fluxes along the profile of salt-stressed *A. donax*. Thus, ALA likely interacted with the methylerythritol 4-phosphate (MEP) pathway and modulate the synthesis of either ABA or isoprene under stressful conditions. Overall, our study highlights the effectiveness of ALA supply through soil fertirrigation in preserving the young apical developing leaves from the detrimental effects of salt stress, thus helping of *A. donax* to cope with salinity and favoring the recovery of the whole plant once the stress is removed.

## 1. Introduction

Climate change is exacerbating the frequency and intensity of processes that cause land degradation. This includes an increase in sea level followed by intrusion of seawater, leading to both soil salinization and a lower quality of freshwater for irrigation [1]. At the same time, the urgent need for expanding biomass energy production and reducing the consumption of fossil fuels without competing with fertile cropland available for agriculture demand use of these marginal soils [2].

The perennial invasive grass *Arundo donax* L., commonly called giant reed, has been recognized among the industrial crops most suitable for biomass production in low-input farming systems and in marginal agricultural areas [3]. *A. donax* is a fast-growing species demonstrated to cope well with increasing salinity due to a sensitive stomatal response (closure) that promptly limits salt uptake by the plant [4]. Salt-stressed *A. donax* also showed enhanced biosynthesis of antioxidants and higher carbohydrates, indicating scavenging of reactive oxygen species (ROS) and osmotic adjustments that may prevent the onset of detrimental effects of salinity [5]. However, elevated salinity hampers both growth and photosynthetic CO_2_ assimilation of *A. donax* plants [6], and the negative effect of salinity on leaf photosynthesis has not yet been clearly disentangled from that on whole-plant growth [7]. Attempts have been carried out to improve *A. donax* growth performance under salt stress though root inoculation with arbuscular mycorrhizae [8].

5-Aminolevulinic acid (ALA) is ubiquitously present into the cells of all living organisms (animals, plants, and microorganisms), being a key intermediate in the biosynthesis of all heterocyclic tetrapyrroles such as heme, porphyrin, chlorophylls, and vitamin B12 [9,10]. In plants, supply of ALA can promote growth and yield by stimulating biosynthesis of pigments [11], photosynthetic CO_2_ assimilation, and nitrogen fixation [12]. Moreover, ALA can prime and mediate plant tolerance against abiotic stress [13], mainly by enhancing the antioxidant capacity to both avoid excessive production of ROS and lower the content of malondialdehyde (MDA) [14,15]. However, the physiological mechanisms through which ALA ameliorates plant tolerance to abiotic stresses are not yet fully understood. In particular, the cross-talk of ALA with plant hormones is still poorly studied, and the relationship between ALA and the emission of volatile organic compound (VOCs) has not been investigated so far. Lack of knowledge about possible beneficial effects of ALA in enhancing plant stress tolerance might limit ALA exploitation in agricultural practices. In the past, supply of ALA was considered an expensive solution to sustainably improve plant performance under stressful conditions. However, recent advances in the bioproduction of ALA by engineered microorganisms [16,17] can make ALA an eco-friendly and inexpensive product suitable for large-scale agricultural applications.

The objective of this study was to test whether performances of *A. donax* under salinity stress can be improved through root exposure to ALA. We tracked the assimilation of mineral elements (Na^+^, Ca^2+^, and K^+^) from the roots throughout the profile of the leaves. Plant biometrical traits and photosynthetic performance, emissions of VOCs, and the concentrations of abscisic acid (ABA) and indole-3-acetic acid (IAA) were assessed in apical, median, and basal leaves during the application of salt stress and following a recovery period.

## 2. Results

### 2.1. Characterization of Arundo donax Control Plants

**Figure 1:**
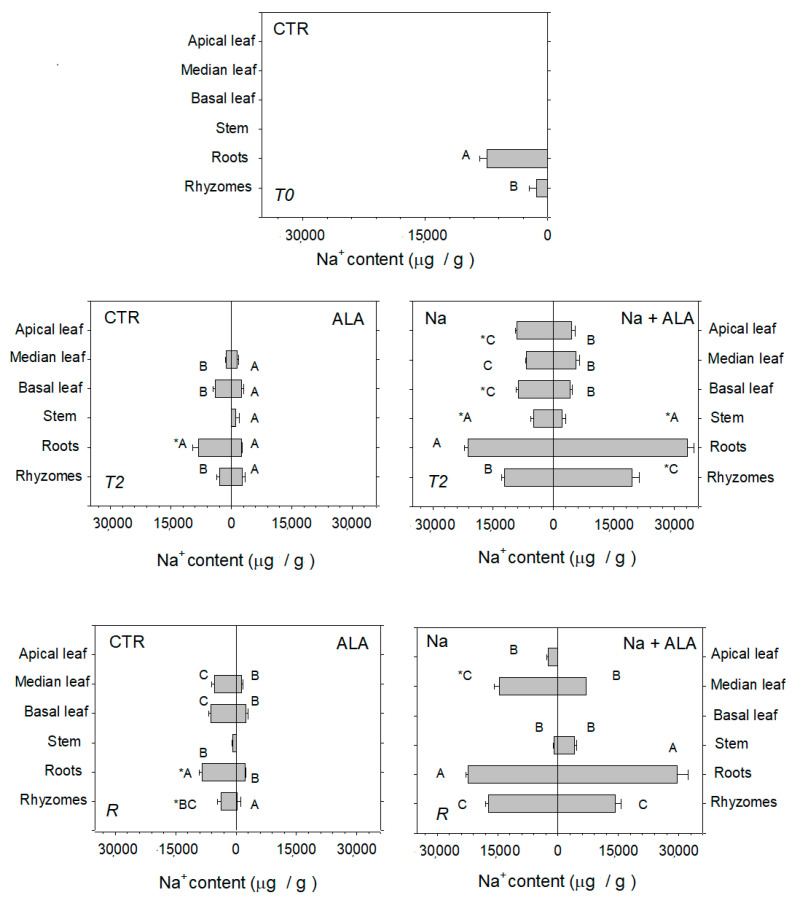
Concentration of Na^+^ (μg/g) measured at different time points (T0, T2, and R) in apical, median, and basal leaves, as well as in stems, roots, and rhizomes of *Arundo donax* controls (CTR), salt-stressed (Na), ALA-treated (ALA), and both salt-stressed and ALA-treated (Na + ALA) plants. Mean values ± SE are shown. Uppercase letters indicate statistically significant differences between (apical, median, and basal) leaves, roots, and the rhizome along the profile of plants undergoing the same treatment; asterisks indicate statistically significant differences between the (apical, median, and basal) leaves, roots, and rhizome of plants undergoing different treatments (*p* < 0.05; *n* = 3). More details about the statistically significant differences between treatments are shown in Table S1.

During the course of the experiment, non-stressed *A. donax* controls progressively developed shorter apical culms and a lower number of internodes (Table 1). A higher Na^+^ concentration was found in the roots than in the rhizome and leaves of *A. donax* controls. Along the plant profile, Na^+^ was higher in the basal than in the median leaves (Figure 1; Table S1). Under optimal nutrient supply, Ca^2+^ concentration increased (although not statistically significant) from the apical to the basal leaves, and it was higher in leaves than in stems and both roots and the rhizome (Table S1). The concentration of K^+^ did not vary among the different leaves along the control plant profile, and it was lower than that in stems but higher than that in both roots and the rhizome (Table S1).

**Figure 2:**
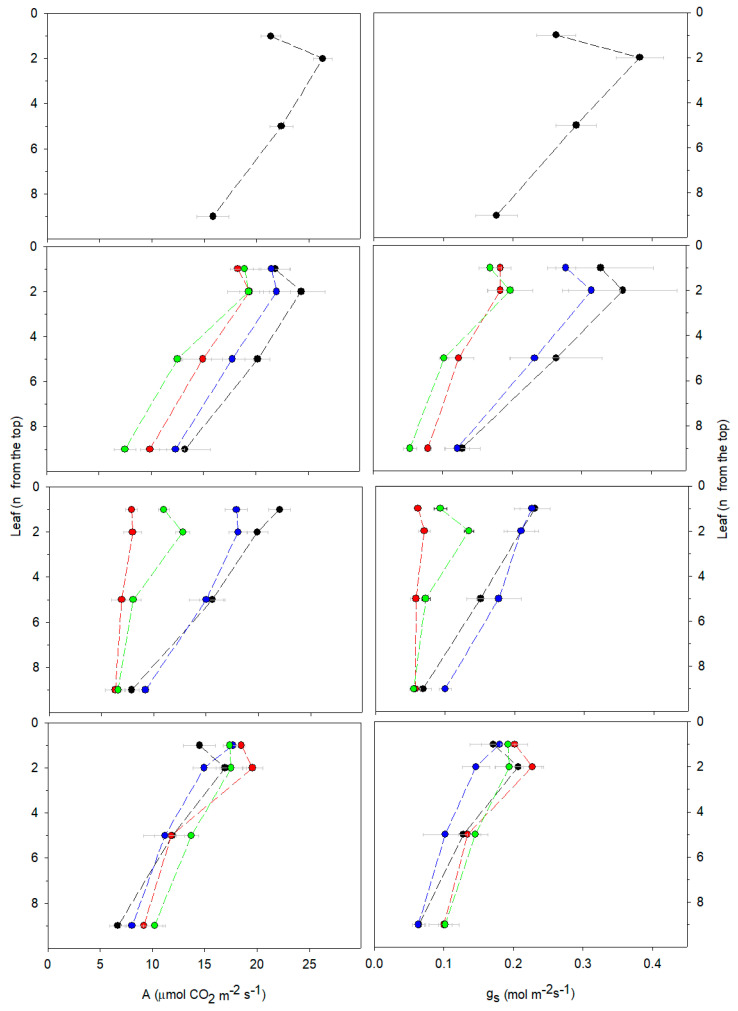
Photosynthetic CO_2_ assimilation rate (**A**) and stomatal conductance (gs), measured at different time points (T0, T1, T2, and R) in the 1st and 2nd (apical), 5th (median), and 9th (basal) leaves counted from the top of *Arundo donax* controls (black circles), salt-stressed (red circles), ALA-treated (blue circles), and both salt-stressed and ALA-treated (green circles) plants. Dotted lines connect leaves belonging to the same plant profile. Mean values ± SE are shown. Statistically significant difference between (apical, median, and basal) leaves of plants undergoing the same treatment and between (apical, median, and basal) leaves of plants undergoing different treatments are shown in Table S2.

Apical leaves of control plants had a higher photosynthetic capacity and stomatal conductance (Figure 2; Table S2), as well as a higher effective quantum yield (ΦPSII) (Table 2) than basal leaves. The chlorophyll concentration of apical control leaves remained lower than that of median leaves and only by the end of the experiment (58 days, R) was it equal to that of basal leaves (Table S3). After 14 days of treatment (T1), apical leaves of control plants had a lower IAA and a higher ABA concentration than basal leaves (Table 3), which resulted in a decreasing ABA/IAA ratio along the plant profile. A lower ALA concentration was found in the apical than in the basal leaves of control plants (Table 4). Over time, the leaf concentration of both IAA and ABA increased, resulting in an equal ABA/IAA ratio along the control plant profile; in particular, the highest concentration of ABA was measured in the basal leaves (Table 3). The ALA concentration also increased during the course of the experiment, nearly becoming equal in all leaves along the profile of control plants (Table 4).

**Figure 3:**
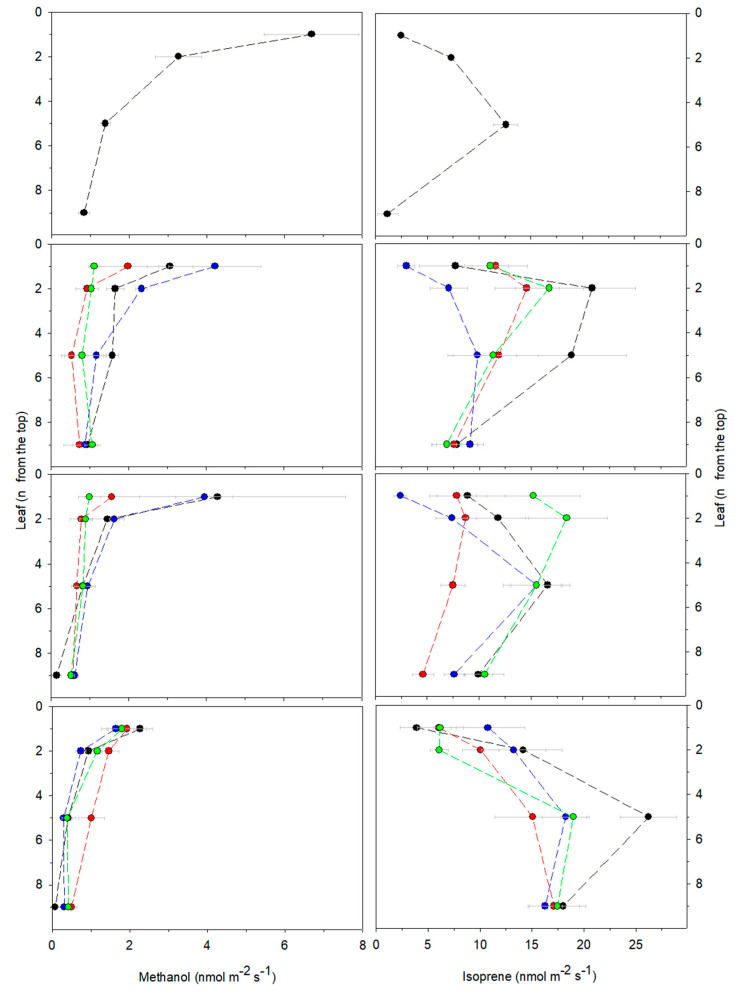
Emission of methanol and isoprene measured at different time points (T0, T1, T2, and R) from the 1st and 2nd (apical), 5th (median), and 9th (basal) leaves counted from the top of *Arundo donax* controls (black circles), salt-stressed (red circles), ALA-treated (blue circles), and both salt-stressed and ALA-treated (green circles) plants. Dotted lines connect leaves belonging to the same plant profile. Mean values ± SE are shown. Statistically significant difference between (apical, median, and basal) leaves of plants undergoing the same treatment and between (apical, median, and basal) of plants undergoing different treatments are shown in Table S4.

During the whole experiment, apical leaves of control plants constitutively emitted higher fluxes of methanol and lower fluxes of isoprene than median and basal leaves (Figure 3; Table S4).

### 2.2. Addition of ALA to Arundo donax Control Plants

Addition of ALA to the nutrient solution of non-stressed *A. donax* enhanced the ALA concentration of leaves along the plant profile, particularly in apical leaves (Table 4). ALA increase did not change the concentration of Na^+^ and Ca^2+^ in the leaves along the plant profile, whereas the concentration of these two elements was reduced the roots with respect to the controls (Table S1). However, the callose concentration in the root tissues remained the same as that measured in the controls (Table S5). Enhanced ALA concentration did not affect plant biometric parameters (Table 1) and did not impact photosynthesis (Figure 2; Table 2 and Table 4) during the whole experiment. However, toward the end of the experiment (after 58 days, R), ALA enhanced the chlorophyll concentration of the apical leaves (Table S3). Continuous addition of ALA prevented the time-dependent increase in IAA reported in controls, first (after 28 days, T2) in the apical and then (after 58 days, R) also in the median and basal leaves (Table 3). Moreover, in all the leaves of ALA-treated plants, the concentration of ABA was first higher (after 28 days, T2) and subsequently lower in the recovery (after 58 days) than in those measured in the controls at the same time points (Table 3). ALA did not affect methanol but reduced, although only temporarily (after 14 days, T1), isoprene emission from all leaves along the *A. donax* plant profile (Figure 3; Table S4).

### 2.3. Response of Arundo donax to Salt Stress and Following Recovery

Salinity stress, after 28 days (at T2), impaired the development of new internodes and the growth of the primary culm (Table 1). At T2, in salt-stressed plants, Na^+^ mainly accumulated in both roots and rhizomes and, to a lesser extent, was equally distributed into the leaves along the plant profile, while the lowest amount of Na^+^ was found in stems (Figure 1; Table S1). Salt stress affected Ca^2+^ accumulation within *A. donax* plants. The Ca^2+^ concentration was higher in apical and lower in basal leaves with respect to the controls. Salinity also reduced the concentration of Ca^2+^ in the stems (Table S1). In salt-stressed *A. donax* plants, the leaf concentration of K^+^ did not change, but K^+^ in roots was lower than that in the controls (Table S1). Prolonged salinity, at T2, dramatically increased the root amount of callose (Table S5).

The onset of salt stress, already after 14 days (at T1), induced stomatal closure in all leaves along the plant profile (Figure 2; Table S2). As salt stress proceeded over time, at T2, stomatal conductance continued to decrease and paralleled the drop in photosynthesis simultaneously measured in apical, median, and basal leaves (Figure 2; Table S2). At T2, stomatal conductance and photosynthesis decreased to the same extent, as shown by unaltered intercellular CO_2_ concentrations in the leaves (Figure 2; Table S2). In salt-stressed *A. donax*, stomatal closure was associated with an increase in ABA and a decrease in IAA (thus resulting in a higher ABA/IAA) both in the apical and median leaves (Table 3). At T2, photosynthesis reduction was further reflected by a lower level of ΦPSII (Table 2) and was also associated with a reduced chlorophyll concentration in median leaves (Table S3). However, even prolonged stressful conditions, at T2, did not impair the maximum quantum efficiency of PSII (Fv/Fm) nor the level of NPQ (Table 2). In addition, prolonged salt stress (at T2) increased the ALA concentration in the median and basal leaves (Table 4). Salt stress conditions reduced the emission of methanol and isoprene from all leaves of *A. donax* plants already at T1. At T2, the reduction of methanol was higher in the apical leaves, while isoprene was more reduced in median and basal leaves (Figure 3; Table S4).

Following 30 days of recovery (at R), the concentration of both Na^+^ and Ca^2+^ decreased in *A. donax* apical leaves, whereas it remained at a similar level to that measured at the end of salt stress (T2) in median leaves. In both roots and rhizomes of recovering plants, Na^+^ remained higher whereas Ca^2+^ was lower than those in the controls. K^+^ accumulation did not change along the entire plant profile (Figure 1; Table S1). Photosynthesis was restored to the levels measured in the controls, especially in the apical leaves, as both stomatal conductance and ΦPSII increased (Figure 2; Table 2; Table S2). A simultaneous decrease in both the ABA and IAA concentration was measured in apical and median leaves recovering from salt stress (Table 3). Nevertheless, the ALA concentration of leaves of plants recovering from salt stress remained higher than that of the controls (Table 4). Recovery enhanced both methanol and isoprene emission from the leaves of salt-stressed plants, and the emission of both methanol and isoprene reached values similar to those in the controls (Figure 3; Table S4).

### 2.4. Effects of ALA Supply in Arundo donax under Salinity and Following Recovery

The addition of ALA did not prevent the growth impairment of the primary culm of *A. donax* and the reduction of internodes induced by salinity (Table 1). ALA treatment enhanced the accumulation of Na^+^ in both roots and the rhizome, although it lowered the accumulation of Na^+^ in the leaves of salt-stressed plants (Figure 1; Table S1). Moreover, ALA treatment increased the Ca^2+^ concentration in the median and basal leaves as well as in the stems, but simultaneously lowered Ca^2+^ in roots and increased the K^+^ concentration in the rhizome of salt-stressed plants (Table S1). In particular, in roots, the addition of ALA prevented the increase in callose concentration induced by salinity (Table S5). Under prolonged salinity conditions (at T2), ALA treatment increased both the stomatal conductance and the photosynthetic CO_2_ assimilation in the apical leaves of salt-stressed plants (Figure 2; Table S3), while maintaining a high ΦPSII (Table 2) and not affecting the chlorophyll concentration (Table S3).

After the application of salt stress (at T1), ALA treatment enhanced the IAA concentration in all leaves and that of ABA only in the apical leaves of salt-stressed plants (Table 3). As salinity proceeded (at T2), ALA treatment decreased the ABA/IAA ratio in the apical leaves of stressed *A. donax* (Table 3). Moreover, the supply of ALA to the roots did not influence methanol, whereas, under pro-longed salt stress (at T2), it sustained isoprene emission from all leaves along the profile of salt-stressed plants to the same rate measured io the controls (Figure 3; Table S4).

After recovery (at R), ALA treatment decreased Na^+^ and increased the Ca^2+^ concentration in the median leaves of salt-stressed plants (Table S1). Moreover, continuous addition of ALA during recovery enhanced IAA and reduced ABA (thus reducing the ABA/IAA ratio) in the apical leaves of salt-stressed *A. donax* (Table 3). However, ALA did not affect photosynthesis (Figure 2; Table S2) or methanol and isoprene emission (Figure 3; Table S4) of salt-stressed plants.

## 3. Discussion

### 3.1. Root Exposure to ALA Affects Element Accumulation, Hormons Balance, Chlorophyls Concentration, and Isoprene Emission in Leaves of Non-Stressed Arundo donax Plants

This is one of the few studies where ALA was supplied to the plants by root exposure [18]. Indeed, in the vast majority of trials conducted so far, ALA has been sprayed onto the leaves [12,13,14]. Our study suggests that, upon absorption by the roots, ALA is translocated homogeneously into the leaves along the profile of *A. donax* plants, where it increasingly accumulates over time. Nevertheless, we cannot exclude that exogenous ALA, either sprayed onto leaves or supplied to roots, also triggers the production of endogenous ALA in different plant parts. Further experiments are needed to assess this possibility (e.g., using labelled ALA).

In our experiments, ALA treatment did not affect the overall uptake of mineral elements into the leaves along the non-stressed *A. donax*, profile and did not affect root callose turnover, which is involved in root development and permeability [19,20]. However, ALA reduced the concentrations of Na^+^ and Ca^2+^ in roots (Table S1). ALA might have stimulated the translocation of both Na^+^ and Ca^2+^ to the leaves, thus limiting their accumulation in belowground plant parts. This could explain the discrepancy between our results and those reported by other studies [21,22,23] in which ALA was sprayed onto the leaves, bypassing element uptake through the roots.

In non-stressed plants, we did not record any significant effect of ALA on leaf photosynthesis or on biometric parameters, consistent with previous studies [24,25,26,27]. Moreover, we showed that ALA treatment did not affect the rate of methanol emission from the leaves of non-stressed *A. donax* plants. Since methanol is released after dimethyl esterification of pectins within the cell walls [28] in actively growing and expanding leaves [29,30], these results further confirm that ALA supply to *A. donax* roots did not affect plant growth and leaf development. The same results indirectly indicate that the amount of ALA supplied to *A. donax* roots did not exert any toxic effects associated with ALA overdosing [31,32]. On the other hand, the stimulation of photosynthesis found in other studies [33,34,35,36,37,38,39,40] could have been due to the method of ALA supply.

In our study, root exposure to ALA induced metabolic changes in non-stressed *A. donax* plants. Indeed, we revealed that ALA influenced with the foliar ABA/IAA balance by maintaining a low IAA level while varying the concentration of ABA. In particular, the effect of ALA on the ABA concentration occurred simultaneously in all leaves along the plant profile, while that on IAA started first from the young developing apical leaves and gradually progressed, over time, toward the basal adult leaves. Although the possibility to manipulate the leaf concentration of both IAA and ABA through ALA supply has already been reported [27], the effects of ALA on the synthesis of endogenous plant hormones are poorly investigated. In fact, the auxin-like activity of ALA in promoting shoot elongation was reported earlier [41], but only recently was it demonstrated that ALA affects auxin biosynthesis and modulates its transport in roots [42]. Our study confirms that ALA can interact with the endogenous synthesis and translocation of IAA in leaves. Moreover, following root exposure to ALA, the foliar ABA concentration of non-stressed *A. donax* was initially enhanced but then decreased with respect to the controls measured at the same time point. Isoprene emission first significantly decreased from all ALA-treated leaves along the plant profile but then returned to the initial rate, while chlorophyll concentration increased only in the apical leaves at the end of the experiment. The synthesis of both ABA and isoprene, and partially chlorophylls, occurs through the same methylerythritol phosphate (MEP) pathway [43], whereas ALA is an intermediate of a tetrapyrrole pathway [44]. Although coordination between MEP and tetrapyrrole pathways allows the regulation and balance of chlorophyll biosynthesis [45], the impact of ALA application on the MEP-derived products has been overlooked to date. In leaves, isoprene emission has already been demonstrated to be directly correlated with the ABA concentration [46]. Our results indicated that ALA may have broken such a relationship in the leaves. We interpret our results as indicating that first (at T1) ALA triggered ABA synthesis at the expense of isoprene emission and then reduced the entire metabolism of the MEP pathway. It is well known that, under non-stressed conditions, the supply of ALA promotes the intermediates of the tetrapyrrole pathway [11,47] and increases the leaf chlorophyll concentration [25,37,38] Consistent with these findings, we observed increased chlorophyll concentrations in non-stressed *A. donax* but only in the apical leaves, which also showed low isoprene emission, at the end of the experiment. As the linear part of the chlorophyll molecule is also made by MEP, the slow and uneven increase in chlorophyll could also be part of a long-term rearrangement of the MEP pathway following root exposure to ALA.

### 3.2. Application of ALA to the Roots Improves the Performance of Salt-Stressed Arundo donax Leaves

Under salinity stress, we measured an increasing ALA concentration in leaves along the *A. donax* longitudinal profile, which supported the involvement of ALA in the response of plants to stress [12,25]. ALA supply counteracts the negative effects of salt stress [15]. Indeed, following the application of salt stress, our results confirmed that *A. donax* stored Na^+^ mainly in the roots and rhizomes [7,48]. We also showed an enhanced biosynthesis of callose in roots that, by altering the cell wall permeability [20,49], may have prevented excessive accumulation of Na^+^, in addition to limiting the absorption of other elements (i.e., Ca^2+^, K^+^) in the roots. The amount of Na^+^ translocated to the leaves was accumulated equally along the salt-stressed *A. donax* longitudinal profile, which is not the case in evergreen dicots [50]. However, root exposure to ALA altered the Na^+^ distribution within *A. donax* by enhancing the accumulation of Na^+^ in the roots while excluding it from the leaves. Since ALA treatment of salt-stressed plants did not affect the leaf K^+^ concentration, it improved the leaf Na^+^/K^+^ ratio, and also prevented a decrease in Ca^2+^ absorbed by salt-stressed plants. ALA-induced changes in the mineral element distribution along the plant profile were associated with either degradation or low synthesis of callose in the roots. Reduced callose could have facilitated the absorption of both Na^+^ and the mineral nutrients (Ca^2+^, K^+^) that mitigated the negative impact of salinity in salt-stressed leaves and after recovery [51]. In addition, the higher root accumulation of Na^+^ in salt-stressed *A. donax* following the supply of ALA might have activated ion transporters that regulate Na^+^ homeostasis and improve tolerance to high Na^+^ levels in roots [52].

The homogeneous accumulation of Na^+^ along the profile of salt-stressed *A. donax* induced the stomata of all leaves to close simultaneously, triggered by an increase in ABA and coupled with a decreased IAA concentration, together with other metabolic changes that have been shown to regulate stomata in response to salt stress [4]. A generalized and rapid stimulation of stomatal closure, occurring already at the early stage of salinity, reduces the transpiration flux of the whole plant and therefore the total amount of Na^+^ (and Cl^−^) delivered to leaves. When combined with the observed preferential accumulation of Na^+^ (and possibly Cl^−^) in the belowground parts of the *A. donax* plants, stomatal closure might have effectively prevented the deleterious effects of excessive accumulation of Na^+^ in the aboveground plant parts, thus enhancing plant tolerance to salinity.

We show that supply of ALA to the roots of salt-stressed *A. donax* prevented the decrease of IAA following salt stress and thus reduced the ABA/IAA ratio, initially in all the leaves along the plant profile and later only in the apical leaves. This result further supported the idea that an interaction between ALA and IAA exists, as also found in the leaves of non-stressed *A. donax* (Table 3). In particular, since IAA can antagonize ABA in mediating stomatal closure [53,54], both a steady IAA level and a low ABA/IAA ratio might explain the increase in stomatal conductance measured in the apical leaves of salt-stressed plants when treated with ALA, which was not associated with a higher leaf concentration of Na^+^ that preferentially accumulated in the roots and rhizomes. In addition, ALA was demonstrated to inhibit ABA-induced stomatal closure by scavenging H_2_O_2_ and Ca^2+^ accumulated in the guard cells [55,56].

Reduction of ΦPSII and the chlorophyll concentration in salt-stressed leaves did not permanently affect photosynthesis, which fully recovered after the stress. Our results highlighted that supply of ALA to the roots increased the photosynthesis of the apical salt-stressed leaves by enhancing stomatal opening and ΦPSII activity. Despite often growing in marginal (salinized) soils, *A. donax* has been shown to be moderately sensitive to salinity [6], which we further confirm to negatively impact growth and photosynthesis. In our experiment, photosynthesis decreased in all the leaves along the profile of salt-stressed plants, while methanol emission started to decrease in young apical leaves. Since methanol fluxes are directly related to processes of plant and leaf growth [29,30,57], our results indicate that whereas salinity impacts photosynthesis systemically at a whole-plant level, the effect on plant growth is gradual and begins from the young developing apical leaves, of which a reduction of their expansion rate has been already documented [58]. Nevertheless, the supply of ALA did not impact the growth or the methanol emission of salt-stressed *A. donax*, thus further indicating that, in our case study, ALA improved photosynthesis without affecting plant growth.

In *A. donax*, the response of isoprene emission to salinity remains ambiguous. In fact, previous studies reported either unaltered [8] or slightly stimulated [4] isoprene emission from salt-stressed *A. donax* plants; however, we measured, toward the end of our experiment, a reduction in isoprene from the median and basal leaves with higher emission rates (Figure 3). A different phenological stage of *A. donax* in which salt stress was applied may explain the various responses of isoprene emission [59]. In fact, we applied salt stress to young developing *A. donax* plants, as indicated by the following parameters measured in non-stressed (control) plants at the beginning compared to the end of the experiment: (a) a sustained methanol emission, especially from apical leaves; (b) a longer length of the apical culm; (c) a higher number of developing internodes. Moreover, since *A. donax* is a perennial plant, environmental conditions occurring before the collection of the rhizomes may have affected the stress response of isoprene emission in the developing plants [60]. Nevertheless, inhibition of isoprene emission under salt-stress conditions has been already documented in *Eucalyptus globulus* and, as in our experiment, isoprene emission returned to the level of non-stressed plants after a recovery period [61]. ALA supply prevented the decrease in isoprene occurring under prolonged salt-stress conditions (at T2) by maintaining the emission at the same level measured in non-stressed plants. This further highlights that the impact of ALA on the MEP pathway changes in response to salt stress. Indeed, isoprene can act as a volatile antioxidant [62] and its sustained emission might also improve the stress tolerance of *A. donax* [63].

## 4. Materials and Methods

### 4.1. Plant Material, Growth Conditions, and Treatments

Rhizomes of *A. donax* were collected in Sesto Fiorentino (Florence, Italy). All rhizomes were kept in tap water for one day and then planted in 6 dm^3^ pots containing quartz sand (one rhizome per pot). From rhizomes, *A. donax* plants were obtained and grown in a climatic chamber under the following controlled environmental conditions: maximum/minimum temperature: 25/16 °C; maximum/minimum relative air humidity: 60/40%; photosynthetic photon flux density (PPFD): 700 μmol m^−2^ s^−1^ for 14 h per day. Before beginning the experiment, plants were regularly watered twice a week with 200 mL of half-strength Hoagland’s solution to replace the exact amount of water transpired from the plant and evaporated from the soil, thus avoiding solution overflow out of the pots [4,5].

Plants were split into four groups of 10 individuals (pots) that underwent different treatments by irrigation with: (1) half-strength Hoagland’s solution (CTR); (2) half-strength Hoagland’s solution enriched with 3 mg L-1 of 5-aminolevulinic acid hydrochloride (ALA, >97%, Merck, Darmstadt, Germany); (3) half-strength Hoagland’s solution enriched with 200 mM of NaCl (Na); (4) half-strength Hoagland’s solution enriched with both 3 mg L-1 of ALA and 200 mM of NaCl (Na + ALA). All solutions were supplied twice a week during the 28-day-long treatment, which was followed by 30 days of recovery. During recovery, half-strength Hoagland’s solution was supplied to all the plants, while ALA- and Na + ALA-treated plants continued to be supplied with ALA.

Sampling and analyses were carried out before starting the treatments (T0), after 14 days (T1) and after 28 days (T2) of treatment, and after the recovery period (R). Since in *A. donax* leaf ontogenesis is closely related to the longitudinal gradient along the plants, measurements were collected from apical new developing leaves, median leaves under development, and basal fully developed leaves. Stems, the rhizome, and roots were also sampled (Figure S1 and details of measured traits below).

### 4.2. Plant Biometric Characteristics and Element Concentration

The apical culm length was measured from the tip of the apical leaf to the first internode, and the number of new internodes developed along the plant profile was assessed in different periods of time: from the development of small plants to the beginning of the experiment (T0); from the beginning to the end of salt and/or ALA application (T2); from the beginning to the end of the recovery period (R).

Dried samples (0.5 g) of apical, median, and basal leaves, culms, rhizomes, and roots were used for mineral element analysis. All samples were microwave-digested at 200 °C in 10 mL of HNO_3_, then the volume was adjusted to 25 mL with distilled water [64], and the concentration of the mineral elements mainly involved in salt stress (Ca^2+^, Na^+^, and K^+^) [7] was determined in triplicate by Flame Atomic Absorption Spectrometry (FAAS, Analyst 200, PerkinElmer, Waltham, MA, USA). Quantification was performed through certified standard solutions for each single element.

### 4.3. Quantification of 5-aminolevulinic Acid (ALA) and Chlorophylls in Leaves

The ALA leaf concentration was measured according to [65]. Frozen leaf samples (0.15 g) were homogenized using liquid nitrogen and then put into 2 mL tubes with 600 µL of 4% trichloroacetic acid (TCA). The extract was centrifuged at 10,000 rpm for 15 min at 4 °C; after that, 0.5 mL of supernatant was mixed with both 500 µL of acid acetic buffer 200 mM (pH 4.6) and 500 µL of acetylacetone and boiled for 10 min. After cooling in ice, 500 µL of Ehrlich’s reagent was added. The absorbance was recorded at 553 nm after static hierarchy for 7 min spectrophotometrically (Varian, Mulgrave, Australia). A calibration curve was constructed using synthetic ALA (Sigma–Aldrich, St. Louis, MO, USA), and the results were expressed as µg g^−1^ leaf fresh weight (FW).

The total amount of chlorophylls was measured according to [66]. Frozen leaf samples (0.25 g) were homogenized in liquid nitrogen and then centrifuged with 80% acetone. The supernatant was used for estimating the total chlorophylls (a + b) by reading absorbance at 663 nm and 643 nm using a ultraviolet–visible (UV/Vis) spectrophotometer and applying the following formula: total chlorophylls (mg/g) = [(12.7 × A663 − 2.69 × A645) × (*v*/*w*)] + [(22.9 × A645 − 4.68 × A663) × *v*/*w*]. where (*v*) is the volume of acetone used and (*w*) the sample weight.

### 4.4. Quantification of Callose in Roots

The callose concentration was analyzed in roots according to [67]. Frozen samples of roots (0.2 g) were homogenized in liquid nitrogen and mixed with 1 mL of pure ethanol (96%) before being stored overnight at −20 °C. Samples were washed three times with 1 mL ethanol 20% containing 5% (*w*/*v*) of polyvinylpolypyrrolidone (PVPP), and 1 mL of 1 M NaOH was added; samples were heated at 85 °C for 20 min to allow callose solubilization. The extract was centrifuged at 11,000 rpm for 20 min at 4 °C, and the supernatant was used for callose assay. The callose assay mixture contained 0.2 mL of supernatant, 0.4 mL of 0.1% (*w*/*v*) aniline blue, 0.21 mL of 1M HCl, and 0.59 mL of 1M glycine–NaOH buffer (pH 9.0). Blanks contained assay mixture lacking aniline blue, and the reaction control lacked a supernatant. The reaction mixture was incubated for 20 min at 50 °C and then for 30 min at room temperature. Curdlan (Sigma–Aldrich, St. Louis, MO, USA) was used as a standard; the absorbance was recorded at 484 nm by a spectrophotometer. For each sample, the absorbance value in the absence of aniline blue was subtracted from the intensity in the presence of aniline blue, and the callose concentration was expressed as µg Curdlan equivalent (CE) g^−1^ fresh weight (FW).

### 4.5. Analysis of Indol-3-acetic Acid (IAA) and Abscisic Acid (ABA)

The concentrations of the free forms of both IAA and ABA were analyzed on the same leaf samples according to [68]. Samples of 0.02 g dry weight (DW) of lyophilized leaf material were extracted with 1 mL isopropanol:acetic acid (95:5, *v*/*v*), and 100 ng of both 13C6-IAA and 2H6-ABA (OlChemIm Ltd., Olomouc, Czech Republic) were added to each sample as internal standards for quantitative mass spectra analysis. After isotope equilibration overnight at 4 °C, the samples were centrifuged for 10 min at 10,000× *g*, and the supernatants were collected. After a double re-extraction of the pellet by using 500 μL of isopropanol:acetic acid (95:5, *v*/*v*) solution, the supernatants were evaporated using a rotary evaporator until fully dry. The residues were suspended with 300 μL methanol and then methylated using diazomethane, before being dried with a N2 stream [69]. Samples were finally re-suspended in 30 μL ethyl acetate, and an aliquot of 2 μL for each sample was injected onto an HP1 capillary column (length 60 m, inner diameter 0.25 mm; film thickness 0.25 μm, Agilent Technologies, Santa Clara, CA, USA) of a gas chromatography–mass spectrometry (GC-MS) system (7890A-5975C, Agilent Technologies, Santa Clara, CA, USA) operating in splitless mode, with helium as a carrier gas at a flow rate of 1 mL min^−1^. The GC injector was set at 280 °C, and the GC oven temperature was first increased from 90–200 °C at a rate of 20 °C min^−1^ and then at a rate of 8 °C min^−1^ until reaching 280 °C. The latter temperature was held for 4 min. The source temperature of the MS was set at 230 °C, and the ionizing voltage was 70 eV. The ions that were monitored were *m*/*z* 130, 136 for the base peak (quinolinium ion), and *m*/*z* 189, 195 for the molecular ion of the methyl-IAA and the methyl-13C6-IAA, respectively; *m*/*z* 190, 194 for the base peak and *m*/*z* 162, 166 for the molecular ion of the methyl-ABA and methyl-2H6-ABA, respectively. The leaf hormone levels were estimated from the corresponding peak area by calculating the ratios between *m*/*z* 130/136 and *m*/*z* 189/195 for IAA and between *m*/*z* 190/194 and *m*/*z* 162/166 for ABA, following the isotope dilution principles [70]. The total amounts of free IAA and ABA were calculated from three replicated measurements.

### 4.6. Gas Exchange and Chlorophyll Fluorescence Measurements

Leaf gas exchange measurements of both CO_2_ and H_2_O coupled to chlorophyll fluorescence were performed by a portable gas exchange system equipped with a fluorometer (Li-Cor 6400, Li-Cor Biosciences Inc., Lincoln, NE, USA). A surface area of 2 cm^2^ was clamped into the cuvette and exposed to a light intensity of 1000 μmol m^−2^ s^−1^, CO_2_ concentration of 400 ppm, temperature of 30 °C, and relative humidity (RH) ranging between 45% and 50% (controlled by an instrument-installed device). After reaching steady-state conditions, the level of photosynthesis (A), stomatal conductance (gs), and internal CO_2_ concentration were calculated according to [71].

The maximal PSII quantum yield (Fv/Fm) was measured in leaves after 30 min of dark-adaptation by applying a saturating pulse, while the effective PSII quantum yield (ΦPSII) and non-photochemical quenching (NPQ) were measured under a light intensity of 1000 μmol m^−2^ s^−1^ [72].

### 4.7. Methanol and Isoprene Emissions

Following gas exchange measurements, a small amount of air (100 mL min^−1^) exiting the cuvette enclosing the leaf was diverted, through a heated (at 50 °C) PFA Teflon^®^ (Colaver SRL, Milano, Italy) sampling line, into a Proton Transfer Reaction—Quadrupole Mass Spectrometer (PTR-QMS) (Ionicon Analytic, Innsbruck, Austria). In PTR-QMS, VOCs with a proton affinity >165 kcal mol^−1^ (i.e., methanol and isoprene) are analyzed through chemical ionization with high-density H_3_O^+^ (produced in an ion source) occurring in a drift tube under constant pressure (2.2 mbar), temperature (50 °C), and electrical field (600 v cm^−2^) conditions, resulting in an ionization energy E/N = 130 Td [73]. In our analysis, the protonated ions with a mass unit (*m*/*z*) = 33 and (*m*/*z*) = 69 related to methanol and isoprene, respectively, were monitored with a dwell time of 1 s for each *m*/*z* for 20 consecutive cycles. During gas exchange and PTR-QMS analysis, a charcoal filter (Supelco, Bellafonte, PA, USA) was placed ahead of the LiCor6400 (Lincoln, NE, USA) in order to remove all VOCs present in the air before reaching the cuvette without affecting the composition or the relative humidity of the air. The methanol and isoprene background was measured from the empty cuvette and then subtracted from the signals recorded after enclosing the leaf. PTR-QMS signals (counts per second) of methanol and isoprene were converted into concentrations (ppbv) by measuring a multi-component gas standard (Apel Riemer, Broomfield, CO, USA) after dilution with VOC-filtered air to achieve a concentration level within the ppbv range.

### 4.8. Statistical Analysis

Descriptive statistics (means, standard errors) of parameters were calculated from the replicated plants under each experimental condition. The Shapiro–Wilk test and the Levene’s test were performed to verify the normality of the data distribution and the homogeneity of variances. Statistically significant differences between the measured variables were analyzed separately at two different levels: (1) within the profile of plants undergoing the same treatment; (2) between plants undergoing different treatments. For each of these two levels, one-way ANOVA followed by post-hoc Tukey’s HSD test was applied. All analyses were performed through the statistical package implemented in Sigma Plot version 12.0 (Systat Software LTD, Hounslow, United Kingdom).

## 5. Conclusions

**Figure 4:**
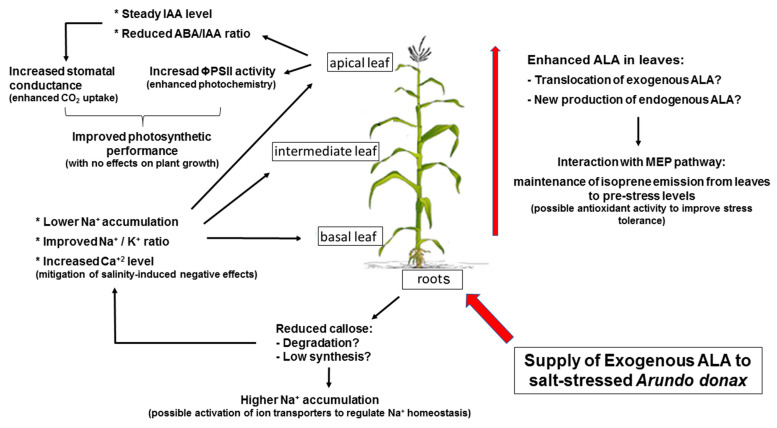
Synthetic overflow of the model functioning of exogenous ALA in salt-stressed *Arundo donax* plants.

Our results show that, following root exposure, ALA increased along the *A. donax* longitudinal profile, suggesting that ALA improves the performance and stress tolerance of young developing leaves (Figure 4). This also favors plant recovery once the stress is removed. Supply of ALA through soil fertirrigation, rather than by foliar spray, may effectively contribute to improve the tolerance to salt stress and, under field conditions, might result in less influence from environmental factors (i.e., light, temperature) that impair ALA stability and its beneficial properties.

## Figures and Tables

**Table 1:** Number of developed internodes and length of the apical culm measured at different time points (T0, T2, and R) in *Arundo donax* controls (CTRL), salt-stressed (Na), ALA-treated (ALA), and both salt-stressed and ALA-treated (Na + ALA) plants. Mean values ± SE are shown. Uppercase letters indicate statistically significant differences between plants undergoing the same treatment compared at different time points, and lowercase letters indicate statistically significant differences between plants undergoing different treatments compared at the same time point (*p* < 0.05; *n* = 3).

		Developed Internodes (*n*)				Apical Culm Length (cm)		
	CTR	Na	ALA	Na + ALA	CTR	Na	ALA	Na + ALA
T0	10.67 ± 0.31 A	-	-	-	5.98 ± 0.15 A	-	-	-
T2	9.00 ± 0.37 B a	1.50 ± 0.37 B b	9.00 ± 0.32 A ac	3.33 ± 1.45 B b	3.47 ± 0.24 B a	2.36 ± 0.31 A b	3.84 ± 0.27 A a	2.99 ± 0.20 A ab
R	5.33 ± 0.49 C b	8.00 ± 0.001 A b	4.60 ± 0.24 B b	7.00 ± 1.00 A b	2.33 ± 0.10 C b	2.54 ± 0.15 A ab	2.38 ± 0.15 B b	3.08 ± 0.23 A a

**Table 2:** Maximum quantum efficiency of photosystem II (F_V_/F_M_), photosystem II quantum yield (ΦPSII), and non-photochemical quenching (NPQ) measured at different time points (T0, T1, T2, and R) in apical, median, and basal leaves along the profile of *Arundo donax* controls (CTR), salt-stressed (Na), ALA-treated ALA (ALA), and both salt-stressed and ALA-treated (Na + ALA) plants. Mean values ± SE are shown. At any time-point, uppercase letters indicate statistically significant differences between (apical, median, and basal) leaves of plants undergoing the same treatment, and lowercase letters indicate statistically significant differences between (apical, median, and basal) leaves of plants undergoing different treatments (*p* < 0.05; *n* = 3).

		*FV/FM*				*ΦPSII*				*NPQ*			
		CTR	Na	ALA	Na + ALA	CTR	Na	ALA	Na + ALA	CTR	Na	ALA	Na + ALA
*T0*	*1st apical*	---	---	---	---	0.296 ± 0.034 A	---	---	---	---	---	---	---
	*2nd apical*	0.829 ± 0.001 A	---	---	---	0.336 ± 0.031 A	---	---	---	1.642 ± 0.065 A	---	---	---
	*median*	0.827 ± 0.002 A	---	---	---	0.283 ± 0.007 A	---	---	---	1.574 ± 0.054 A	---	---	---
	*basal*	0.824 ± 0.002 A	---	---	---	0.217 ± 0.036 A	---	---	---	1.727 ± 0.100 A	---	---	---
*T1*	*1st apical*	---	---	---	---	0.309 ± 0.033 A a	0.298 ± 0.028 A a	0.283 ± 0.023 A a	0.292 ± 0.014 A a	---	---	---	---
	*2nd apical*	0.831 ± 0.002 A a	0.815 ± 0.008 A ab	0.824 ± 0.002 A ab	0.807 ± 0.004 A b	0.344 ± 0.035 A a	0.314 ± 0.018 A a	0.286 ± 0.023 A a	0.298 ± 0.028 A a	1.550 ± 0.062 A a	1.611 ± 0.094 A a	1.505 ± 0.119 A a	1.461 ± 0.246 A a
	*median*	0.827 ± 0.001 A a	0.816 ± 0.006 A a	0.813 ± 0.003 A a	0.821 ± 0.003 A a	0.290 ± 0.013 AB a	0.255 ± 0.024 AB a	0.246 ± 0.027 AB a	0.222 ± 0.021 AB a	1.691 ± 0.070 A a	1.611 ± 0.043 A a	1.752 ± 0.083 A a	1.748 ± 0.041 A a
	*basal*	0.821 ± 0.003 A a	0.815 ± 0.004 A a	0.812 ± 0.006 A a	0.812 ± 0.003 A a	0.198 ± 0.027 B a	0.182 ± 0.013 B a	0.186 ± 0.004 B a	0.147 ± 0.028 B a	1.659 ± 0.057 A ab	1.653 ± 0.020 A ab	1.462 ± 0.051 A b	1.704 ± 0.062 A a
*T2*	*1st apical*	---	---	---	---	0.309 ± 0.011 A a	0.168 ± 0.010 A b	0.289 ± 0.009 A a	0.232 ± 0.012 A b	---	---	---	---
	*2nd apical*	0.795 ± 0.008 A b	0.915 ± 0.032 A a	0.838 ± 0.026 A ab	0.903 ± 0.032 A ab	0.288 ± 0.017 AB a	0.158 ± 0.006 A b	0.289 ± 0.018 A a	0.236 ± 0.012 A a	1.792 ± 0.076 A a	1.763 ± 0.104 A a	1.683 ± 0.075 A a	1.839 ± 0.072 A a
	*median*	0.860 ± 0.022 B a	0.944 ± 0.009 A a	0.889 ± 0.016 AB a	0.910 ± 0.025 A a	0.250 ± 0.013 B a	0.147 ± 0.008 AB b	0.255 ± 0.008 A a	0.117 ± 0.006 B b	1.824 ± 0.074 A a	1.850 ± 0.054 A a	1.785 ± 0.130 A a	1.860 ± 0.033 A a
	*basal*	0.951 ± 0.003 C a	0.955± 0.006 A a	0.945± 0.006 B a	0.928 ± 0.019 A a	0.162 ± 0.002 C a	0.116 ± 0.012 B b	0.166 ± 0.011 B a	0.162 ± 0.010 B a	2.081 ± 0.067 A a	1.909 ± 0.128 A a	1.727 ± 0.064 A a	1.676 ± 0.190 A a
*R*	*1st apical*	---	---	---	---	0.251 ± 0.004 AB b	0.292 ± 0.004 A a	0.304 ± 0.013 A a	0.274 ± 0.010 A ab	---	---	---	---
	*2nd apical*	0.868 ± 0.034 A a	0.887 ± 0.021 A a	0.904 ± 0.006 AB a	0.946 ± 0.001 A a	0.279 ± 0.019 A a	0.292 ± 0.010 A a	0.264 ± 0.009 A a	0.253 ± 0.019 A a	1.727 ± 0.066 A a	1.821 ± 0.176 A a	1.578 ± 0.058 A a	1.554 ± 0.013 A a
	*median*	0.894 ± 0.001 A c	0.871 ± 0.002 A b	0.923 ± 0.002 A a	0.904 ± 0.003 B b	0.231 ± 0.002 B a	0.210 ± 0.039 AB a	0.215 ± 0.015 B a	0.223 ± 0.010 AB a	1.745 ± 0.108 A a	1.568 ± 0.097 A a	1.607 ± 0.037 A a	1.486 ± 0.026 A a
	*basal*	0.910 ± 0.022 A a	0.876 ± 0.010 A ab	0.880 ± 0.010 B a	0.818 ± 0.005 C b	0.168 ± 0.008 C a	0.199 ± 0.027 B a	0.181 ± 0.013 B a	0.201 ± 0.014 B a	1.828 ± 0.052 A a	1.827 ± 0.166 A a	1.922 ± 0.183 A a	1.795 ± 0.043 B a

**Table 3:** Concentration of indol-3-Acetic Acid (IAA) and abscisic acid (ABA) (pmol/g FW) measured at different time points (T1, T2, and R) in apical, median, and basal leaves along the profile of *Arundo donax* controls (CTR), salt-stressed (Na), ALA-treated (ALA), and both salt-stressed and ALA-treated (Na + ALA) plants. Mean values ± SE are shown. At any time-point, uppercase letters indicate statistically significant differences among (apical, median, and basal) leaves of plants undergoing the same treatment, and lowercase letters indicate statistically significant differences between (apical, median, and basal) leaves of plants undergoing different treatments (*p* < 0.05; *n* = 3).

		CTR			Na			ALA			Na + ALA		
		IAA	ABA	ABA/IAA	IAA	ABA	ABA/IAA	IAA	ABA	ABA/IAA	IAA	ABA	ABA/IAA
*T1*	apical	346.1 ± 26.3 B b	1216.3 ± 59.0 A b	3.5 ± 0.4 A a	295.4 ± 41.7 B b	2030.3 ± 96.2 A bc	6.9 ± 1.3 A b	618.7 ± 109.1 A a	1586.6 ± 130.7 A b	2.6 ± 0.7 A a	614.3 ± 76.0 B a	3184.5 ± 118.6 A a	5.2 ± 0.7 A b
	*median*	299.6 ± 25.9 B b	804.6 ± 97.1 B a	2.7 ± 0.6 AB a	321.7 ± 32.0 B b	1129.5 ± 98.1 B a	3.5 ± 0.7 AB b	210.9 ± 68.6 B b	714.4 ± 213.3 B a	3.4 ± 2.1 A a	1953.7 ± 252.6 A a	1281.8 ± 257.6 B a	0.7 ± 0.2 B b
	*basal*	678.3 ± 76.0 A b	614.8 ± 55.7 B a	0.9 ± 0.2 B a	954.9 ± 56.0 A bc	1249.2 ± 150.0 B a	1.3 ± 0.2 B a	523.4 ± 46.3 AB b	797.0 ± 206.8 A a	1.5 ± 0.5 A a	2250.3 ± 127.4 A a	1540.2 ± 77.3 B a	0.7 ± 0.1 B b
*T2*	apical	1002.9 ± 123.4 A a	1184.5 ± 149.6 B b	1.1 ± 0.3 A a	552.0 ± 75.4 A b	2015.5 ± 235.6 A a	3.7 ± 0.9 A b	484.6 ± 73.1 A b	1440.5 ± 247.0 B a	3.0 ± 1.0 A a	849.1 ± 47.4 A ab	2168.9 ± 183.3 A a	2.6 ± 0.4 A a
	*median*	666.9 ± 57.7 A a	1127.7 ± 206.1 B b	1.7 ± 0.5 A a	498.3 ± 80.6 A a	1406.1 ± 142.0 A ab	2.8 ± 0.7 A b	757.1 ± 76.6 A a	1894.7 ± 65.9 B a	2.5 ± 0.3 A b	529.1 ± 32.0 B a	1088.6 ± 223.5 B b	2.1 ± 0.5 A a
	*basal*	901.1 ± 193.1 A a	1981.4 ± 60.7 A b	2.2 ± 0.5 A b	-	-	-	520.0 ± 64.0 A a	3076.1 ± 117.8 A a	5.9 ± 1.0 A a	-	-	-
*R*	apical	625.7 ± 86.9 B a	1698.5 ± 112.9 A ac	2.7 ± 0.6 A b	280.0 ± 36.0 B c	1767.8 ± 87.9 A a	6.3 ± 1.1 A a	368.0 ± 46.9 A b	1185.2 ± 43.9 A b	3.2 ± 0.5 A ab	305.1 ± 27.4 A b	829.9 ± 110.2 A b	2.7 ± 0.6 A b
	*median*	600.6 ± 175.4 B a	994.3 ± 73.9 B a	1.7 ± 0.6 A ab	396.0 ± 13.1 A ab	964.4 ± 337.5 B a	2.4 ± 0.9 A ab	481.7 ± 22.3 A ab	624.2 ± 68.6 B a	1.3 ± 0.2 B b	154.9 ± 10.3 B b	689.0 ± 39.8 B a	4.4 ± 0.6 A a
	*basal*	1254.9 ± 141.1 A a	1978.4 ± 92.8 A a	1.6 ± 0.3 A a	-	-	-	451.4 ± 33.1 A b	1051.1 ± 32.2 A b	2.3 ± 0.2 AB a	-	-	-

**Table 4:** Concentration of 5-aminolevulinic acid (ALA) (µg/g) measured at different time points (T0, T2, and R) in apical, median, and basal leaves of *Arundo donax* controls (CTR), salt-stressed (Na), ALA-treated (ALA), and both salt-stressed and ALA-treated (Na + ALA) plants. Mean values ± SE are shown. At any time-point, uppercase letters indicate statistically significant differences between (apical, median, and basal) leaves of plants undergoing the same treatment, and lowercase letters indicate statistically significant differences between (apical, median, and basal) leaves of plants undergoing different treatments (*p* < 0.05; *n* = 3).

		CTR	Na	ALA	Na + ALA
*T0*	*apical*	0.65 ± 0.19 B	---	---	---
	*median*	1.51 ± 0.35 AB	---	---	---
	*basal*	2.37 ± 0.51 A	---	---	---
*T2*	*apical*	1.35 ± 0.33 A b	2.03 ± 0.29 C b	3.76 ± 0.32 A a	4.20 ± 0.42 A a
	*median*	2.05 ± 0.30 A b	4.04 ± 0.15 B a	3.60 ± 0.70 A a	4.21 ± 0.35 A a
	*basal*	1.82 ± 0.21 A b	6.03 ± 0.45 A a	3.59 ± 0.39 A c	4.68 ± 0.24 A ac
*R*	*apical*	3.50 ± 0.32 A b	5.06 ± 0.49 A a	5.08 ± 0.77 A a	5.52 ± 0.20 B a
	*median*	3.96 ± 0.55 A b	6.93 ± 0.64 A a	6.17 ± 0.45 A a	7.68 ± 0.48 A a
	*basal*	3.96 ± 0.26 A b	---	6.71 ± 0.40 A a	---

## Data Availability

The data presented in this study are available on request from the corresponding author.

## Supplementary Materials

The following are available online at https://www.mdpi.com/article/10.3390/ijms23084311/s1.
