# Supplementary material for: Root Exposure to 5-Aminolevulinic Acid (ALA) Affects Leaf Element Accumulation, Isoprene Emission, Phytohormonal Balance, and Photosynthesis of Salt-Stressed Arundo donax
Source: Int J Mol Sci. 2022 Apr 13;23(8):4311. doi: 10.3390/ijms23084311 (PMC9028702; doi:10.3390/ijms23084311)
Supplement: Supplementary file 1 [file ijms-23-04311-s001.zip › ijms-1645512-supplementary.pdf]

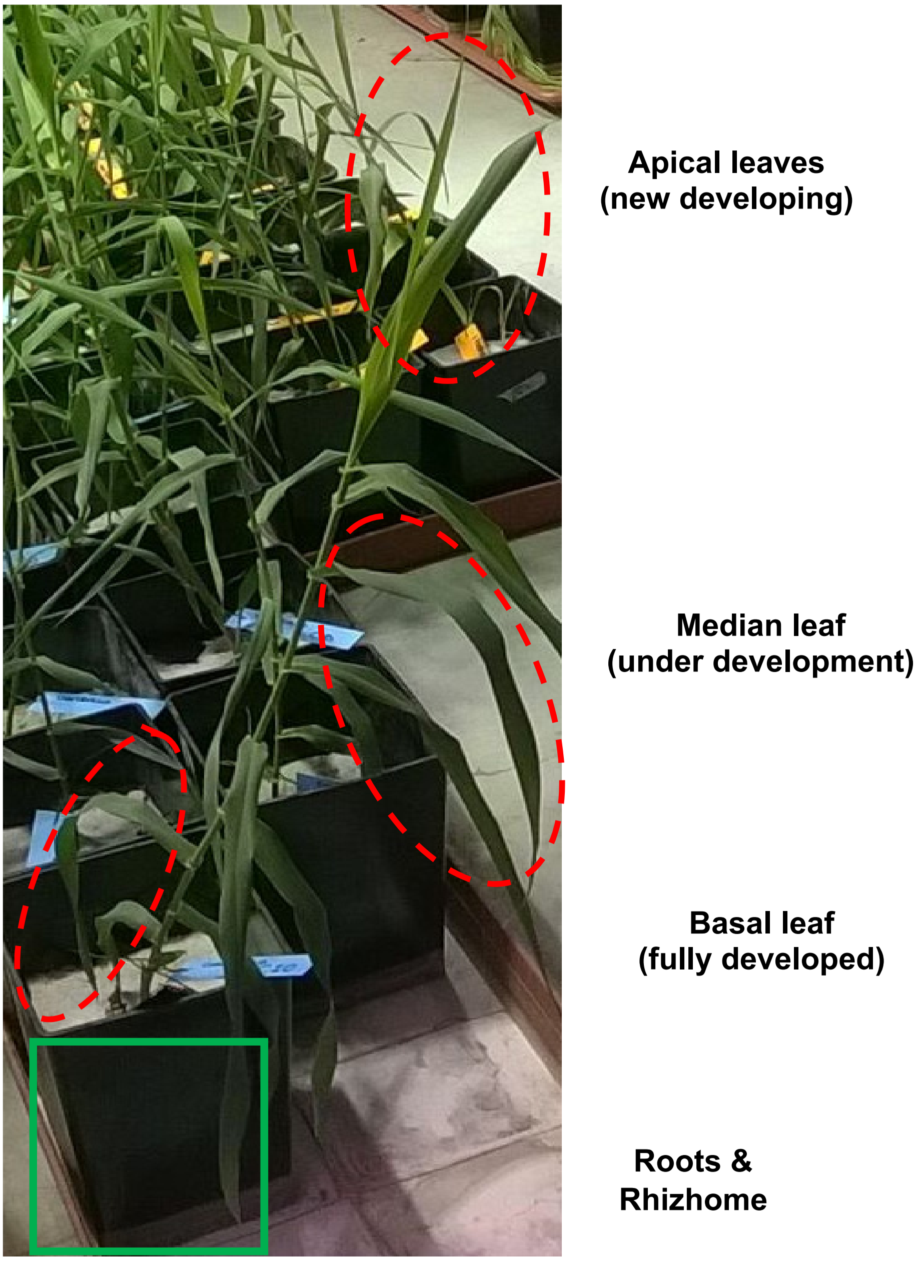

Figure S1 - Picture of *Arundo donax* plant profile indicating the position of apical, median and basal leaves, as well as the pot containing the roots and the rhizome.

	Na+				Ca^{+2}				K^+			
	**CTR**	**Na**	**ALA**	**Na + ALA**	**CTR**	**Na**	**ALA**	**Na + ALA**	**CTR**	**Na**	**ALA**	**Na + ALA**
T0												
Apical leaf	n.d.	---	---	---	2562.4 ± 391.8 AC	---	---	---	49935.5 ± 3202.4 B	---	---	---
Median leaf	n.d.	---	---	---	3846.3 ± 169.4 A	---	---	---	45057.1 ± 4983.2 B	---	---	---
Basal leaf	n.d.	---	---	---	4068.9 ± 498.8 A	---	---	---	44195.7 ± 4758.8 B	---	---	---
Stems	---	---	---	---	1347.8 ± 147.2 C	---	---	---	57691.9 ± 1455.8 A	---	---	---
Roots	7459.7 ± 831.4 A	---	---	---	1907.3 ± 568.2 C	---	---	---	14923.5 ± 748.1 C	---	---	---
Rhizome	1359.6 ± 879.1 B	---	---	---	544.2 ± 37.0 BC	---	---	---	14816.0 ± 985.3 C	---	---	---
T2												
Apical leaf	----	9024.3 ± 500.5 C a	---	4520.4 ± 857.0 B b	2735.7 ± 337.4 D b	6364.2 ± 667.1 A a	1844.8 ± 669.8 BC b	6957.7 ± 71.3 C a	42085.8 ± 1747.3 C a	37007.5 ± 3700.1 C a	34626.1 ± 1567.8 A a	33428.0 ± 876.8 AC a
Median leaf	1284.5 ± 189.7 B b	6756.9 ± 146.5 CD a	1392.8 ± 399.9 A b	5540.5 ± 833.7 B a	7153.8 ± 373.5 C a	6382.5 ± 198.1 A a	7957.1 ± 917.6 A a	7975.0 ± 218.6 C b	42722.8 ± 4559.2 C a	43068.0 ± 3385.8 AC a	45338.0 ± 1957.3 A a	36827.2 ± 3612.2 AC a
Basal leaf	3939.4 ± 644.5 B b	8829.3 ± 501.8 C a	2464.7 ± 623.4 A b	4055.1 ± 538.3 B b	8957.3 ± 268.5 A a	5438.0 ± 594.3 A b	9112.6 ± 1022.5 A a	9006.5 ± 780.8 A a	36123.7 ± 2733.5 AC a	44650.0 ± 1204.9 C a	40931.5 ± 4316.4 A a	40695.9 ± 5596.7 AC a
Stems	---	4993.6 ± 647.1 D a	1108.6 ± 789.7 A b	2171.9 ± 909.6 B b	612.7 ± 106.2 B a	294.1 ± 19.9 B b	613.8 ± 33.5 B a	566.3 ± 5.2 B a	40147.6 ± 2495.5 C a	48749.5 ± 1270.7 AC a	33184.9 ± 1737.0 AC a	43448.5 ± 3982.4 A a

	Roots	8194.6 ± 1406.9 A c	21242.5 ± 848.7 A d	2562.3 ± 91.0 A b	33302.6 ± 1601.8 A a	2874.9 ± 14.0 D a	2615.0 ± 425.9 C a	1240.3 ± 336.2 BC b	945.0 ± 114.0 B b	19114.0 ± 662.4 B a	6402.6 ± 300.0 B b	21957.1 ± 2225.0 B a	9716.5 ± 1716.1 B b
	Rhizome	2966.6 ± 696.5 B b	12264.9 ± 740.5 B c	2658.8 ± 751.4 A b	19445.8 ± 1879.8 C a	3979.1 ± 123.4 D a	753.8 ± 9.4 C b	333.6 ± 99.1 C b	505.4 ± 112.1 B b	9290.7 ± 506.4 B b	9294.1 ± 510.0 B b	13129.4 ± 31.2 B b	27059.3 ± 2394.5 C a
R	*Apical leaf*	n.d.	2474.3 ± 360.5 B	n.d.	n.d.	1547.9 ± 472.7 B a	509.6 ± 44.9 BC a	709.6 ± 145.2 B a	686.4 ± 82.8 B a	44218.9 ± 4197.2 A a	43342.0 ± 2866.4 A a	41991.9 ± 5897.4 A a	44963.7 ± 2322.8 AC a
	Median leaf	5381.8 ± 676.1 C bc	14516.9 ± 1207.0 C a	2376.4 ± 190.3 B b	6910.2 ± 19.9 B c	5986.7 ± 306.4 C ab	4566.4 ± 228.5 A b	4033.5 ± 504.6 A b	8209.3 ± 921.5 A a	26564.5 ± 2258.2 BC a	32623.4 ± 2279.8 A a	29241.0 ± 2792.3 A a	30334.2 ± 4782.8 AC a
	Basal leaf	6174.6 ± 599.5 C a	---	4344.7 ± 440.9 B a	---	8875.4 ± 268.5 A a	---	5212.6 ± 295.8 A b	---	35033.3 ± 3236.0 AC a	---	30423.2 ± 4554.9 AB a	---
	Stems	896.5 ± 127.8 B b	929.3 ± 304.4 B a	---	4051.6 ± 521.2 B a	437.7 ± 60.8 B a	520.3 ± 127.4 BC a	367.1 ± 41.2 B a	437.1 ± 53.7 B a	18152.1 ± 3519.2 B a	36080.7 ± 3643.2 A a	21176.8 ± 3701.0 B a	35405.5 ± 2822.7 A a
	Roots	8424.7 ± 739.7 A b	22414.7 ± 516.1 A a	2170.1 ± 2037 B c	29668.1 ± 2631.9 A a	3780.4 ± 550.6 D a	1348.6 ± 297.7 B b	1685.6 ± 376.7 B b	1100.2 ± 355.6 B b	25488.5 ± 2683.7 BC a	16046.3 ± 3319.8 B a	17599.3 ± 252.8 B a	15640.5 ± 2206.9 BC a
	Rhizome	3787.4 ± 877.7 BC c	17292.1 ± 627.7 C a	6380.8 ± 1004.1 A a	14264.2 ± 1360.7 C a	4666.6 ± 15.5 B a	349.2 ± 121.9 C b	427.5 ± 39.9 B b	609.9 ± 165.3 B b	13322.8 ± 823.0 B a	11150.6 ± 890.5 B a	15472.7 ± 2680.0 B a	10781.1 ± 4243.2 B a

Table S1 – Concentration (μg / g) of mineral elements (Na^+; Ca^{+2}; K^+) measured at different time points (T0, T2 and R) in the apical, median, and basal leaves as well as in stems, roots and rhizome of *Arundo donax* controls (CTR), salt-stressed (Na), ALA-treated (ALA) and both salt-stressed and ALA-treated (Na + ALA) plants. Mean values ± SE are shown. At any time point, uppercase letters indicate statistically significant differences between (apical, median and basal) leaves, roots and rhizome of

plants undergoing the same treatment; lowercase letters indicate statistically significant differences between the (apical, median and basal) leaves, roots and rhizome of plants undergoing different treatments ($P < 0.05$; n = 3).

	A (µmol m^{-2} s^{-1})				gs (µmol m^{-2} s^{-1})				Ci (ppm)			
	**CTR**	**Na**	**ALA**	**Na + ALA**	**CTR**	**Na**	**ALA**	**Na + ALA**	**CTR**	**Na**	**ALA**	**Na + ALA**
T0 1st apical	21.35 ± 0.96 A	---	---	---	0.262 ± 0.028 A	---	---	---	228 ± 9 A	---	---	---
2nd apical	26.33 ± 0.89 A	---	---	---	0.328 ± 0.034 A	---	---	---	248 ± 8 A	---	---	---
median	22.37 ± 1.10 A	---	---	---	0.291 ± 0.029 AB	---	---	---	235 ± 10 A	---	---	---
basal	15.83 ± 1.53 B	---	---	---	0.176 ± 0.030 B	---	---	---	203 ± 14 A	---	---	---
T1 1st apical	21.86 ± 1.37 A a	18.28 ± 0.71 A a	21.50 ± 1.77 A a	18.93 ± 1.39 A a	0.327 ± 0.076 A a	0.183 ± 0.015 A b	0.202 ± 0.018 AC a	0.168 ± 0.016 A a	248 ± 31 A a	202 ± 12 A a	244 ± 9 A a	188 ± 5 A a
2nd apical	24.33 ± 2.26 A a	19.42 ± 0.95 A a	22.00 ± 1.29 A a	19.32 ± 1.99 A a	0.359 ± 0.078 A a	0.183 ± 0.018 A b	0.227 ± 0.016 A a	0.197 ± 0.033 A a	246 ± 40 A a	197 ± 13 A a	250 ± 15 A a	207 ± 18 A a
median	20.18 ± 1.19 AB a	14.93 ± 1.89 AB a	17.75 ± 1.97 AB ab	12.51 ± 0.40 B b	0.263 ± 0.066 A a	0.123 ± 0.022 AB b	0.134 ± 0.014 BC ab	0.076 ± 0.009 B b	234 ± 25 A ab	172 ± 14 A b	245 ± 10 A a	176 ± 10 A ab
basal	13.22 ± 2.41 B a	9.88 ± 0.91 B a	12.34 ± 0.87 B a	7.50 ± 1.02 B a	0.128 ± 0.026 A a	0.079 ± 0.005 B b	0.100 ± 0.022 B a	0.081 ± 0.006 B a	207 ± 2 A a	176 ± 22 A a	207 ± 9 A a	136 ± 32 A a
T2 1st apical	19.90 ± 1.00 AC a	7.89 ± 0.54 A b	17.90 ± 1.06 A a	10.97 ± 0.52 A a	0.209 ± 0.025 A a	0.061 ± 0.004 A b	0.225 ± 0.026 A a	0.093 ± 0.009 C c	215 ± 13 A ac	171 ± 3 A b	239 ± 9 A a	185 ± 9 A bc
2nd apical	22.03 ± 1.05 A a	8.00 ± 0.85 A c	18.06 ± 0.87 A a	12.79 ± 0.67 A b	0.229 ± 0.022 A a	0.070 ± 0.008 A b	0.209 ± 0.020 A a	0.134 ± 0.007 A c	212 ± 18 A a	194 ± 10 A a	229 ± 9 A a	208 ± 7 A a
median	15.60 ± 1.22 C a	6.94 ± 0.94 A b	15.02 ± 1.65 A a	8.06 ± 0.75 B b	0.151 ± 0.020 AB ac	0.058 ± 0.008 A b	0.177 ± 0.032 AB a	0.072 ± 0.006 BC c	205 ± 11 A a	188 ± 10 A a	230 ± 12 A a	198 ± 6 A a
basal	7.90 ± 0.70 B a	6.35 ± 0.98 A a	9.23 ± 0.32 B a	6.61 ± 0.32 B a	0.068 ± 0.012 B ab	0.057 ± 0.008 A b	0.100 ± 0.009 B a	0.055 ± 0.004 B b	187 ± 22 A a	200 ± 7 A a	226 ± 10 A a	186 ± 7 A a
R 1st apical	14.50 ± 1.54 A a	18.50 ± 0.31 AB a	17.68 ± 0.46 A a	17.38 ± 0.63 A a	0.171 ± 0.034 A a	0.202 ± 0.018 AC a	0.180 ± 0.010 A a	0.192 ± 0.010 A a	237 ± 11 A a	222 ± 11 A a	214 ± 7 A a	227 ± 7 A a
2nd apical	16.93 ± 2.09 A a	19.58 ± 0.97 A a	14.95 ± 1.10 AC a	17.53 ± 1.09 A a	0.207 ± 0.034 A a	0.227 ± 0.016 A a	0.146 ± 0.020 A a	0.194 ± 0.020 A a	242 ± 6 A a	233 ± 6 A ab	209 ± 7 A b	226 ± 6 A ab
median	11.90 ± 0.36 AB a	11.81 ± 2.63 BC a	11.20 ± 0.98 BC a	13.73 ± 0.68 AB a	0.128 ± 0.007 A a	0.134 ± 0.014 BC a	0.102 ± 0.014 AB a	0.145 ± 0.008 AB a	230 ± 6 A ab	237 ± 8 A a	200 ± 10 A b	224 ± 5 A ab
basal	6.71 ± 0.77 B a	9.19 ± 1.81 C a	8.05 ± 1.10 B a	10.21 ± 1.02 B a	0.064 ± 0.064 A a	0.100 ± 0.020 B a	0.063 ± 0.022 B a	0.102 ± 0.010 B a	215 ± 3 A a	228 ± 7 A a	200 ± 11 A a	214 ± 9 A a

Table S2 – Photosynthetic CO_2 assimilation arte (A); stomatal conductance (gs), intercellular CO_2 concentration (Ci) measured at different time points (T0, T1, T2 and R) in (1st, 2nd) apical, median and basal leaves of *Arundo donax* controls (CTR), salt-

stressed (Na), ALA-treated (ALA) and both salt-stressed and ALA-treated (Na + ALA) plants. Mean values ± SE are shown. At any time point, uppercase letters indicate statistically significant differences between (apical, median and basal) leaves of plants undergoing the same treatment, and lowercase letters indicate statistically significant differences between (apical, median and basal) leaves of plants undergoing different treatments ($P < 0.05$; n = 3).

	CTR	Na^+	ALA	Na^+ + ALA
T0				
apical	1.47 ± 0.21 A	---	---	---
median	1.64 ± 0.01 A	---	---	---
basal	---	---	---	---
T2				
apical	0.87 ± 0.05 B a	0.91 ± 0.16 A a	0.83 ± 0.08 A a	0.83 ± 0.07 A a
median	1.38 ± 0.03 A a	0.82 ± 0.09 A b	1.28 ± 0.11 A a	0.87 ± 0.08 A b
basal	1.30 ± 0.08 A a	---	1.27 ± 0.18 A a	---
R				
apical	0.86 ± 0.05 B b	0.89 ± 0.03 B bc	1.08 ± 0.01 A a	0.75 ± 0.03 B b
median	1.33 ± 0.06 A a	1.14 ± 0.03 A ab	1.14 ± 0.09 A ab	0.88 ± 0.03 A b
basal	0.82 ± 0.05 B a	---	0.93 ± 0.08 A a	---

Table S3 – Total chlorophylls concentration (mg / g) measured at different time points (T0, T2 and R) in apical, median and basal leaves of *Arundo donax* controls (CTR), salt-stressed (Na), ALA-treated (ALA) and both salt-stressed and ALA-treated (Na + ALA) plants. Mean values ± SE are shown. At any time point, uppercase letters indicate statistically significant differences between (apical, median and basal) leaves of plants undergoing the same treatment, and lowercase letters indicate statistically significant differences between (apical, median and basal) leaves of plants undergoing different treatments ($P < 0.05$; n = 3).

	Methanol (nmol m^{-2} s^{-1})				Isoprene (µmol m^{-2} s^{-1})			
	**CTR**	**Na**	**ALA**	**Na + ALA**	**CTRL**	**Na**	**ALA**	**Na + ALA**
T0 1*st* apical	6.70 ± 1.22 A	---	---	---	2.47 ± 0.43 B	---	---	---
2nd apical	3.27 ± 0.60 B	---	---	---	7.33 ± 1.25 C	---	---	---
median	1.39 ± 0.12 B	---	---	---	12.59 ± 1.16 A	---	---	---
basal	0.84 ± 0.15 B	---	---	---	9.09 ± 1.02 AC	---	---	---
T1 1*st* apical	3.06 ± 0.59 A a	1.98 ± 0.79 A b	4.22 ± 1.19 A a	1.11 ± 0.15 A a	7.69 ± 3.45 B a	11.57 ± 3.02 A a	2.97 ± 0.81 B b	11.07 ± 1.74 A a
2nd apical	1.65 ± 0.23 B ab	0.93 ± 0.29 A b	2.33 ± 0.15 AB a	1.04 ± 0.03 A b	20.86 ± 4.18 A a	14.57 ± 3.09 A ab	7.06 ± 1.78 AB b	16.72 ± 3.29 A ab
median	1.58 ± 0.15 B a	0.53 ± 0.19 A a	1.17 ± 0.22 B a	0.80 ± 0.54 A a	18.87 ± 5.29 A a	11.87 ± 1.69 A a	9.81 ± 2.37 AB b	11.34 ± 4.35 A a
basal	0.94 ± 0.21 B a	0.73 ± 0.41 A a	0.87 ± 0.32 B a	1.06 ± 0.19 A a	7.84 ± 1.98 A a	7.57 ± 2.22 A a	9.11 ± 1.30 A b	6.86 ± 1.39 A a
T2 1*st* apical	4.29 ± 3.29 A a	1.56 ± 0.71 A b	3.95 ± 0.74 A a	0.99 ± 0.29 A a	8.89 ± 3.64 A a	7.84 ± 1.89 A a	2.43 ± 0.61 B b	15.23 ± 4.49 A a
2nd apical	1.45 ± 0.43 A a	0.78 ± 0.29 A b	1.63 ± 0.12 B a	0.89 ± 0.16 A a	11.83 ± 2.96 A b	8.71 ± 1.06 A b	7.38 ± 1.52 B b	18.44 ± 3.95 A a
median	0.81 ± 0.10 A a	0.66 ± 0.14 A a	0.94 ± 0.21 B a	0.82 ± 0.23 A a	16.61 ± 1.27 A a	7.48 ± 1.14 A b	15.24 ± 2.50 A a	15.54 ± 3.21 A a
basal	0.14 ± 0.12 A b	0.56 ± 0.07 A ab	0.61 ± 0.10 B a	0.51 ± 0.10 A ab	9.92 ± 1.38 A ab	4.60 ± 1.03 A b	7.60 ± 0.96 B ab	10.53 ± 1.86 A a
R 1*st* apical	2.26 ± 0.16 A a	1.93 ± 0.67 A a	1.64 ± 0.23 A a	1.80 ± 0.36 A a	3.88 ± 1.58 B a	6.00 ± 1.69 B a	10.77 ± 3.56 A a	6.17 ± 2.19 B a
2nd apical	0.94 ± 0.26 B a	1.46 ± 0.27 A a	0.74 ± 0.13 B a	1.17 ± 0.11 AB a	14.15 ± 3.74 AB a	10.06 ± 1.72 AB a	13.24 ± 3.12 A a	6.07 ± 0.90 B a
median	0.41 ± 0.20 B a	1.01 ± 0.34 A a	0.30 ± 0.06 B a	0.39 ± 0.14 B a	26.22 ± 4.72 A a	15.06 ± 3.65 AB a	18.24 ± 2.25 A a	18.99 ± 1.27 A a
basal	0.80 ± 0.16 B a	0.50 ± 0.15 A a	0.31 ± 0.06 B a	0.42 ± 0.08 B a	18.02 ± 0.30 A a	17.10 ± 2.46 A a	16.26 ± 1.67 A a	17.46 ± 2.74 A a

Table S4 – Emission of methanol and isoprene measured at different time points (T0, T1, T2 and R) in (1st, 2nd) apical, median and basal leaves of *Arundo donax* controls (CTR), salt-stressed (Na), ALA-treated (ALA) and both salt-stressed and ALA-treated (Na + ALA) plants. Mean values ± SE are shown. At any time point, uppercase letters indicate statistically significant differences between (apical, median and basal) leaves of plants undergoing the same treatment, and lowercase letters indicate statistically significant differences between (apical, median and basal) leaves of plants undergoing different treatments (P < 0.05; n = 3).

	callose (μg / g)
**CTR**	45.70 ± 5.24 c
**Na^+**	145.52 ± 5.36 a
**ALA**	47.44 ± 2.25 c
**Na^+ + ALA**	10.38 ± 1.28 b

Table S5 – Callose concentration measured in roots of *Arundo donax* controls (CTR), salt-stressed (Na), ALA-treated (ALA) and both salt-stressed and ALA-treated (Na + ALA) after the application of salt stress (at T2). Mean values ± SE are shown and lowercase letters indicate statistically significant differences between plants undergoing different treatments ($P < 0.05$; n = 3).
